# Comparison of different methods of more effective chest compressions during cardiopulmonary resuscitation (CPR) in the dental chair

**DOI:** 10.1016/j.resplu.2022.100286

**Published:** 2022-08-10

**Authors:** Takashi Hitosugi, Norimasa Awata, Yoichiro Miki, Masanori Tsukamoto, Takeshi Yokoyama

**Affiliations:** a Section of Dental Anesthesiology, Division of Maxillofacial Diagnostic & Surgical Sciences, Faculty of Dental Science, Kyushu University, 3-1-1 Maidashi, Higashi-ku, Fukuoka City, Fukuoka 812-8582, Japan; b Smile Dental Clinic, 1-1-5 Kamimoto-cho-nishi, Chuo-ku, Osaka City, Osaka 542-0062, Japan; c School of Interdisciplinary Science and Innovation, Faculty of Arts and Science, Kyushu University, 744 Motooka, Nishi-ku, Fukuoka-shi, Fukuoka 812-8582, Japan; d Department of Dental Anesthesiology, Kyushu University Hospital, 3-1-1 Maidashi, Higashi-ku, Fukuoka City, Fukuoka 812-8582, Japan

**Keywords:** Cardiopulmonary resuscitation (CPR), Manual chest compression, Dental chair, Stool position, Dental, CPR, Cardiopulmonary resuscitation, ERC, European Resuscitation Council, AHA, American Heart Association

## Abstract

**Introduction:**

When performing cardiopulmonary resuscitation (CPR) on a patient who has suffered a cardiopulmonary arrest during dental treatment, few dental chairs have sufficient stability to perform effective chest compressions. We previously proposed a method of stabilizing the backrest of a dental chair using a support stool. As a result, we confirmed that the vertical displacement of the backrest could be significantly reduced. In the present study, we verified the effectiveness of the stool stabilization method using several dental chairs (flat and curved) with significantly different backrest shapes.

**Methods:**

Vertical displacement of the backrests of dental chairs was recorded. Data were obtained at three different stool positions (without a stool, under the chest at the level that participants were performing manual chest compressions, and under the shoulders). Reduction displacement ratios were calculated to evaluate the effectiveness of the stool positions.

**Results:**

The method significantly reduced the vertical displacement of the backrest for all types. When the curvature of the backrest was large, the reduction in vertical displacement was 40% when the stool was placed under the chest at the level of manual chest compressions and 65% when placed underneath the shoulder. In the case of a flat dental chair, this reduction was 90% when using a stool in either position, compared to no stool.

**Conclusion:**

When we need to perform CPR on a patient in the dental chair, placing a stool under the shoulders allows effective manual chest compression by firmly supporting the backrest of a dental chair of any shape.

## Introduction

The positioning of a stool underneath a dental chair to stabilize it in order to perform an effective manual chest compression site has been reported previously.1., 2. This technique is intended to provide greater stability by reclining the dental chair and placing a stool under the backrest so that it is in contact with the back of the chair. It was adopted in the 2015 and 2021 European Resuscitation Council (ERC) guidelines.3., 4. However, we found that the support effect varied depending on the position of the stool. For dental chairs with a heavily curved back outline, a stool placed under the shoulder compared to under the manual chest compression site significantly reduced vertical displacement, resulting in a large reduction ratio.^2^ However, that study was based on only one type of dental chair.

The purpose of this study was to compare the effectiveness of three stool positions (no stool, under the manual chest compression, and under the shoulder) in stabilizing three types of dental chairs with significantly different external shapes.

## Methods

### Ethical statements

This was a manikin study and there were no human participants. The Institutional Review Board at Kyushu university confirmed that no ethical approvals were required.

### Study design and setting

Three dental chairs (#1: EOMαⅡ®; GC, Tokyo, Japan, #2: EOM-PLUS SS®; GC, Tokyo, Japan, #3: EOM ∑®; GC, Tokyo, Japan) were used in this study. #1 exterior has a severely curved backrest exterior, #2 and #3 have a flatter shape.

The study procedure was performed according to a previously established method in which the CPR manikin (Resusci Anne® Torso Basic version 2011; Laerdal Medical AS) was positioned on the reclined dental chair with the upper end of the manikin torso aligned with the top edge of the backrest in Fig. 11., 2.. The superior surface of the backrest under the lower half of the manikin’s sternum was positioned horizontally using a leveling instrument. The hand position for manual chest compressions was the center of the chest. A metal indicator (point P) was secured to the inferior surface of the dental chair directly under the area for manual chest compressions and made parallel to the floor with a level gauge. The distance of point P relative to the inferior surface of the backrest remained fixed for the duration of the study. Displacement of point P was captured using video recordings and measured while each health care provider performed manual chest compressions on the resuscitation manikin. Manual chest compressions depth was kept between 5.1 to 6.0 cm during the study. The actual manual chest compressions depth was evaluated with the manikin‘s Skill-Reporter system which has a green light that indicates manual chest compressions depths of 5.1 to 6.0 cm. Any compressions outside of that range (i.e., without the green light) were excluded. When compression depths were within 5.1 to 6.0 cm, the vertical displacement of the backrest from its initial position was recorded and included for analysis. Video data were transferred to a computer, and the backrest vertical displacement measurements were determined using the simultaneously captured ruler for reference. The stabilizing stool placed under the backrest of the dental chair for this study was round with a hard seating surface (diameter 30 cm; height 45 cm; FB-01ALLBK, Fuji Boeki Co., ltd.). The superficial edge of the stool’s seat was set to vertically contact the backrest either just under the area for manual chest compressions or under the shoulders.

**Fig. 1:**
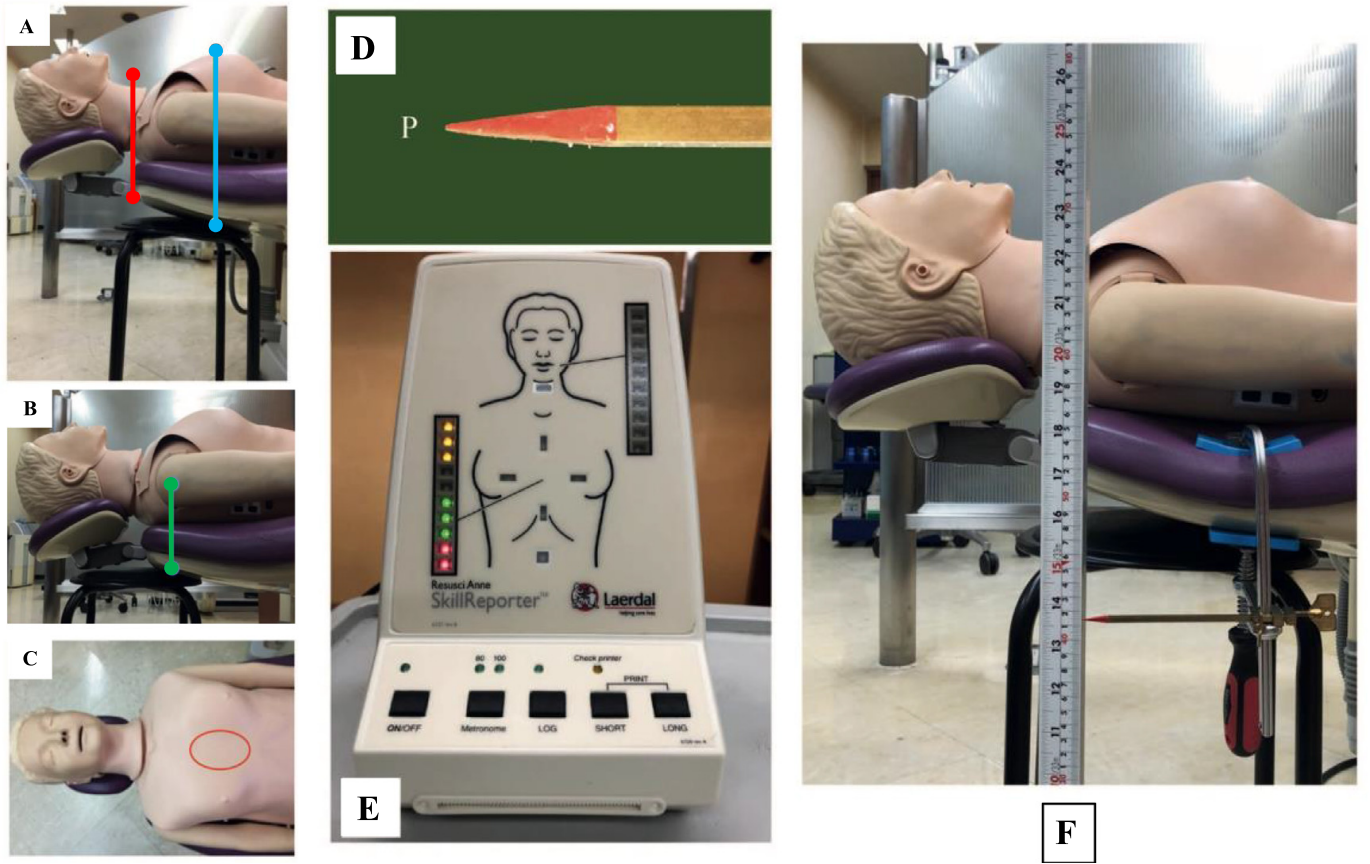
Manikin setup and positioning of the stabilizing stool. The upper end of the manikin torso was aligned with the top edge of the backrest (A; red line). The superior surface of the backrest under the lower half of the manikin sternum was positioned horizontally using a leveling instrument. The edge of the stool’s seating surface was set to touch the backrest vertically under the area for manual chest compressions (A; blue line). The stool was set to touch the backrest vertically under the shoulders (B; green line). The center of the manikin’s chest (C; red ellipse) was the hand position during chest compressions. Measuring vertical displacement and chest compression depth. A metal indicator (D: point P) was made parallel to the floor with a leveling gauge and secured to the inferior surface of the dental chair directly under the area for manual chest compressions next to a fixed vertical-measurement instrument. Chest compression depth was assessed using the manikin’s Skill-Reporter system (E) with green lights indicating chest compression depths of 5.1–6.0 cm and red lights for 3.8–5.0 cm. The distance of point P relative to the inferior surface of the backrest remained fixed (F).

### Outcomes

Three American Heart Association-trained BLS providers (A: 47-year-old male, 175 cm, 93 kg; B: 44-year-old male, 177 cm, 60 kg; C: 44-year-old female, 157 cm, 50 kg.) were tested at their convenience. Each study participant individually performed 10 contiguous rounds of manual chest compressions (20 compressions per round; 200 compressions total) at a pace of 100 compressions per minute in synchrony with a metronome for each of the 3 stool configurations (i.e., under manual chest compressions area, under shoulders, or no stool). Participants rested sufficiently after each round to avoid fatigue. A total of 600 manual chest compressions 5.1 to 6.0 cm in depth were recorded per participant in each dental chair. Each participant had a total of 1,800 manual chest compressions recorded at three different dental chairs.

### Analyses and statistics

The programming language R (version 4.0.2; The Comprehensive R Archive Network, USA) was used for statistical analysis. Displacement measurements (maximum distance of Point P from baseline during MCC) by three participants were analyzed separately at each of the three respective positional configurations. The change in vertical displacement for each of the two stool positions (under manual chest compressions or under the shoulders) compared with no stool position (baseline) was calculated using the following equation (1).

(1) $$reduction\;\;ratio\;=\;1\;-\;\frac{\;displacement\;\;with\;\;stool}{\;displacement\;\;without\;\;stool}$$

Data sets were analyzed using the Shapiro-Wilk test to determine the normality of distribution. The non-parametric data sets were then analyzed using the Wilcoxon rank sum test to determine statistical significance (P < 0.001).

## Results

**Fig. 2:**
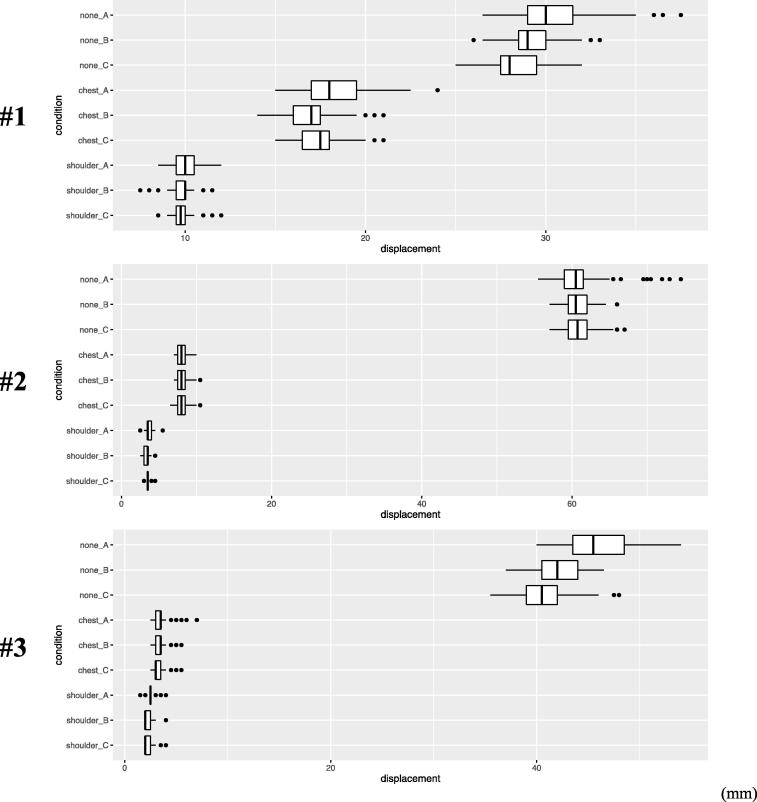
Vertical Displacement Measurements. The physiques of the Provider's body size (A, B, C) who performed manual chest compression differed greatly. The thick lines represent median values, the boxes represent interquartile ranges, over and underlines represent date ranges, and circles represent outliers. In dental chairs #2 and #3, there were significant differences in stool positions because between under the shoulders and under the chest. In contrast #1 made little difference. (Please note that the horizontal axis does not start with zero only in the alpha figure).

**Fig. 3:**
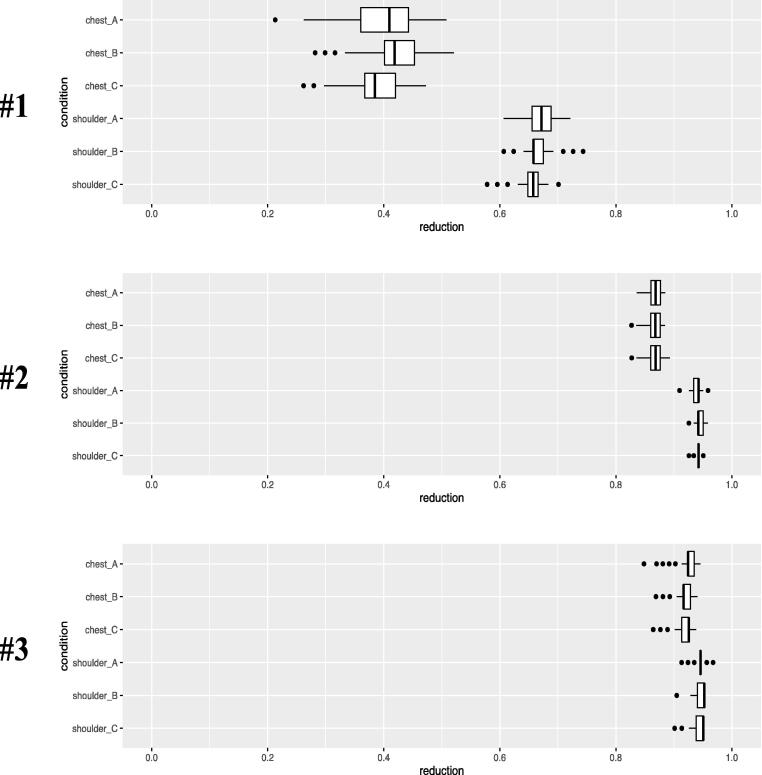
Calculated Reduction Ratios. Measurements of dental chair backrest vertical displacement during manual chest compressions and calculated reduction ratios. Results are expressed as median (interquartile range).

The vertical displacements of the dental chair backrest induced by manual chest compressions were assessed with and without the place of a stool (placed under manual chest compressions or under the shoulders). A total of 5400 (1800 per participant) manual chest compressions were recorded, but 32 were excluded due to an unclear recording or inappropriate compression depth. The vertical displacement was significantly reduced, and the reduction rate increased in all situations when using the stool as a stabilizer (Fig. 2). The model of dental chairs has different characteristics for supportive effect depending on the position of the stool. Particularly in chair #1 which has a severely curved backrest exterior, there was a significant difference in stool positions. As we placed a stool under manual chest compressions in #1, Compared to no stool, a stool underneath the manual chest compression site resulted in a median reduction ratio in chair displacement of 0.41, 0.42, and 0.38 for the three BLS providers, and a stool underneath the shoulder resulted in a median reduction ratio in chair displacement of 0.67, 0.65, and 0.64 (Fig. 3). In contrast chairs, #2 and #3 had flatter shapes. They made little difference between under the shoulders and under manual chest compressions. However, these were clearly different from the no stool (Fig. 2, 3).

## Discussion

This study has shown that manual chest compressions can be effectively performed by placing a stool under the shoulder for any shape of the dental chair when we need to perform CPR on a patient in a dental chair.

**Fig. 4:**
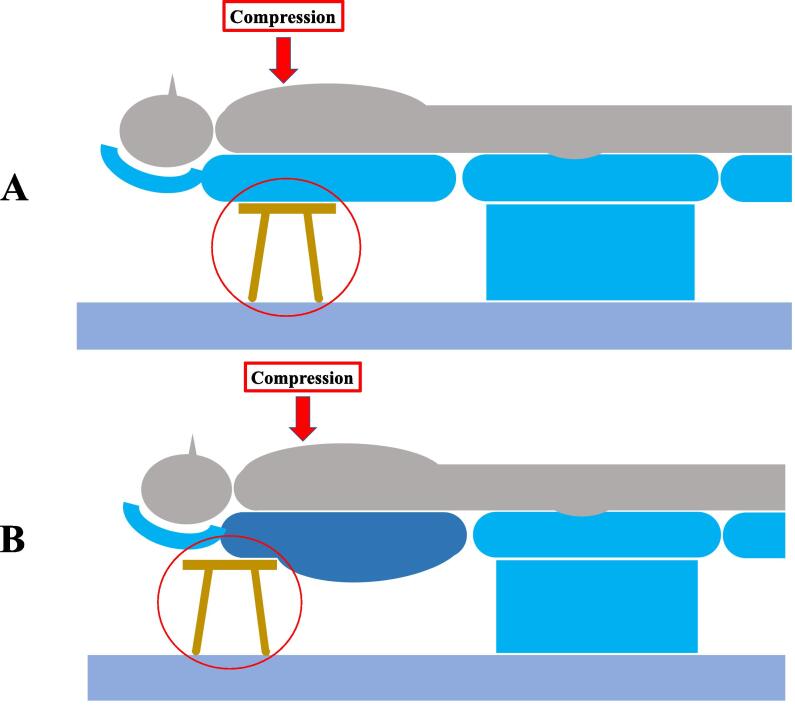
Differences in stool position for manual chest compressions (Red arrow). A: The stool was placed under the chest compression site. B: The stool was placed under the shoulders. When the backrest of the dental chair was curved in shape as shown in B, placing the stool on the shoulder side reduced the vertical displacement.

When a patient suffers a cardiopulmonary arrest during dental treatment, many practitioners are uniformly upset and impatient. This is because they are not skilled in emergency life-saving procedures and have difficulty calmly determining what to do next and reacting smoothly; the ERC and AHA guidelines emphasize the importance of minimizing interruptions during manual chest compressions. If cardiac arrest is suspected, manual chest compressions should be initiated as soon as possible in a stable location.5., 6. However, if it is difficult to quickly and safely move a patient seated in a dental chair to the floor, CPR for a patient seated in a dental chair may be less effective than manual chest compressions if the vertical displacement of the backrest is large. Additionally, the backrest may not be adequately supported for effective resuscitation.7., 8., 9., 10. Many modern dental chairs are ergonomically designed with a curved backrest that gradually flattens upward to the patient's shoulders; the external shape of the chair backrest is on the lower bodyside (e.g., abdomen and lower back), rather than on the area where manual chest compressions are performed, is often more pronouncedly curved, and for stabilization adversely affect the point of contact with the stool, reducing stability (Fig. 4). The exterior of many current dental chairs is characterized by an upward more flattened profile. If the stabilizing stool is positioned below the shoulders, it provides a larger, wider contact area and is more easily stabilized. The back of the dental chair is further stabilized when there is a distance between the patient's seating position and the stabilizing stool.

The video image results showed little displacement of the stool in response to the external force of the chest compressions. When performing this method of CPR in practice, we determined that no other person was needed to stabilize the stool position.

## Limitations

This study had some limitations. First, only three types of dental chairs were included in this study; however, many types of dental chairs are in use worldwide. Second, the usefulness of other types of stabilizers has not yet been tested, and the stools were only set in specific positions. Additional studies should be conducted to evaluate other positions of the stabilizing stools for maximum effectiveness. Third, this study did not consider the effect of the dental chair cushion or the use of a hard backboard during manual chest compressions.11., 12. Next, we need to demonstrate the clinical effectiveness of the method. For example, we should study subjects to improve the success rate of chest compression depth and reduce fatigue from chest compressions. Finally, because this study was conducted using a mannequin model, caution should be exercised in applying the results to human patients. However, no previous studies have demonstrated that vertical displacement is significantly reduced using this technique in several types of dental chairs. The placement of a stool to support the dental chair is a simple and effective way to increase the effectiveness of manual chest compressions and should be utilized when performing CPR on a patient sitting in a dental chair.

## Conclusion

The present study demonstrates that our method is more stable for effective manual chest compressions in any dental chair if the stool is positioned under the shoulders rather than the manual chest compressions. These results suggest that dentists need to prepare in advance an appropriate method (stool position) according to the external shape of the dental chair backrest to perform effective manual chest compressions and that it is very effective.

## Declaration of Competing Interest

The authors declare no competing interests.

## CRediT authorship contribution statement

**Takashi Hitosugi:** Conceptualization, Methodology, Writing – original draft, Writing – review & editing. **Norimasa Awata:** Data curation. **Yoichiro Miki:** Software, Validation, Investigation, Writing – review & editing. **Masanori Tsukamoto:** Supervision. **Takeshi Yokoyama:** Conceptualization, Methodology, Writing – review & editing, Project administration, Investigation, Supervision.
